# Forensic age estimation in the living by 0.31 Tesla low-field MRI of the distal radius

**DOI:** 10.1007/s00414-025-03705-w

**Published:** 2026-01-02

**Authors:** Christian Ottow, S. Schmidt, R. Schulz, L. Sottmann, W. Heindel, A. Helfen, T. Krähling, H. Pfeiffer, A. Schmeling, V. Vieth

**Affiliations:** 1 Clinic for Radiology, University of Münster and University Hospital Münster, Albert-Schweitzer-Campus 1, Münster, 48149 Germany; 2 Institute of Legal Medicine, University of Münster and University Hospital Münster, Röntgenstraße 23, Münster, 48149 Germany; 3 Present Address: Clinic for Radiology and Neuroradiology, Klinikum Ibbenbüren, Große Straße 41, Ibbenbüren, 49477 Germany

**Keywords:** Age determination by skeleton, Forensic medicine, Bone development, Wrist, Magnetic resonance imaging

## Abstract

**Objectives:**

Examination of the radius’ distal epiphysis by means of low-field MRI in order to find a reliable method to correctly assess majority in both sexes.

**Materials & methods:**

650 volunteers of German nationality, evenly distributed to groups of 25 per sex and years of proven age in the age bracket of 12–24 years, were examined between 2021 and 2023 in a single center, prospective, cross-sectional setting. A 0.31 T dedicated joint scanner was used, acquiring a proton density-weighted (PDw) sequence in Dixon technique (Dixon) in coronal slice orientation yielding a fat-sensitive water-suppressed (fat-only) and a water-sensitive fat-suppressed (water-only) series. A classification was formulated for assessment and tested against the proven age. The relevant statistics were defined, the intra- and interobserver-agreements were determined, and the differences between the sexes were analyzed.

**Results:**

The minimum age for stage 6 of the classification was found to be 18.42 years in male individuals and 17.25 years in female individuals. A Mann-Whitney-U Test implies significant sex-related differences for stage 3 (*p* < 0.01) and stage 4 (*p* < 0.01), but not for stage 2 (*p* < 0.162), stage 5 (*p* < 0.193) and stage 6 (*p* < 0.146). The intra- and interobserver-agreement levels were substantial.

**Conclusion:**

When using the presented setting of a low-field 0.31 T dedicated joint scanner, acquiring PDw Dixon fat-only and water-only series of the radius’ distal epiphysis and using the presented classification, majority can be determined in male but not in female individuals of our cohort. Therefore, 0.31 T low-field MRI yields similar opportunities as examinations by means of high-field MRI scanners.

## Introduction

Since the advent of magnetic resonance imaging (MRI) over four decades ago, or even longer depending on whether one considers the first conducted MRI scan or the clinical introduction, all fields of medicine found use for this imaging technique free of ionizing radiation [1]. However, certain areas of specialized interest still wait to benefit from the nowadays established technology, due to different limiting factors. One such field is applied forensic age estimation in the living, resulting from the dauntingly high requirements that must be met before the implementation of novel methods, as it regularly deals with questions of substantial relevance. In this context, the international and multidisciplinary study group on forensic age diagnostics (AGFAD) [5, 17, 18], has not yet been enabled to implement MRI into its framework of recommended examinations for cases in which the surpassing of a defined age limit is to be checked with the highest standard of proof. In these cases, the “minimum-age concept” has to be used, meaning that for each recommended method the chronological age of the youngest individual of the method’s referential study who shows the same developmental stage as the examined individual is assumed to be the individual’s minimum age. Though this method does not provide a precise actual age, it will almost always lead to a lower estimated age compared to the actual age and should prevent an overestimation of the chronological age and subsequently a falsely attested majority. Obviously, minimum ages of reliable reference studies are of the utmost importance in applied forensic age assessment. Aside from a visual inspection of the individual’s physique with anamnesis, the currently recommended examinations include the acquisition of an orthopantomogram (OPG), the acquisition of a projectional radiography of the left hand and in case of a finished development of the hand’s skeletal structures, the additional acquisition of a computed tomography (CT) of the clavicles’ medial epiphyses [17].

Recently, an approach by Vieth et al. [19] using high-field MRI of 3.0 Tesla (T), including a dedicated high-field classification, has demonstrated a potentially viable approach by examination of the distal femur’s epiphyses. Additionally, further studies showed, that along with others [2–4, 6–10, 14, 20], this approach may also be extended and applied to the epiphyses of other long bones, such as the distal radius’ epiphysis [13]. However, using high-field scanners of 1.5 T and 3.0 T puts applied age estimation in competition with the scanners’ clinical use, requires elaborate infrastructure and features high energy consumption and thusly the implementation appears limited.

The study group consequently decided to explore the use of contemporary low-field joint scanners of 0.31 T, in order to circumvent the aforementioned conflicts and to evaluate the viability of such an approach in applied age estimation in the living, after initial feasibility studies had proven successful [12], while also providing a referential study.

## Materials and methods

### Study population

A total of 325 male and 325 female volunteers between 12 and 24 years of German nationality were enrolled between 2021 and 2023 via advertisements in newspapers, flyers and social media. The groups were each composed of 25 participants per year of age and sex and filled consecutively determined by the applicants’ submission date. Since the study aimed at examining the physis’ development in healthy individuals, the inclusion criteria were only composed of known sex and proven age. Further noted characteristics of the volunteers were known illnesses as well as past and present medication. The exclusion criteria were developmental diseases and disorders, injuries to the knee joint area, in order to prevent the inclusion of individuals with hitherto unknown adverse post-traumatic changes to the physiological skeletal maturation, and common contra-indications of low-field MRI, especially incorporated metal elements and potential gravidity. Noteworthy, due to the construction of the scanner claustrophobia was no exclusion criterion. This study received approval by the responsible ethics committee and a signed written informed consent from each volunteer or in case of minors from the legal guardian was obtained. The study’s setting is prospective and the examined volunteers’ data was used to establish the dataset for the project “*age estimation in refugees of questionable minority*” that was funded by the European Asylum, Migration and integration Fund (AMIF) and the German Federal Office for Migration and Refugees.

### Scanner and sequences

The MRI-scans were performed with a 0.31 T scanner (Esaote O-Scan Premium, Esaote S.p.A.) using a dedicated dual phased array (DPA) coil for the wrist. A proton density-weighted (PDw) spin echo (SE) sequence in Dixon technique (Dixon, sequence trade name SPED PD), in coronal orientation was acquired (TR 1800 ms; TE 20 ms / 31 ms; duration 6:16 min; slice thickness 3.5 mm; measured matrix 288 × 288; measured pixel size 0,56 × 0,56 mm²; reconstructed matrix 512 × 512; reconstructed pixel size 0,31 × 0,31 mm²) from which fat-sensitive water-suppressed (fat-only) and water-sensitive fat-suppressed (water-only) series were computed.

For the morphological assessment of the stages the images were viewed on a PACS-workstation with simple post processing allowed i.e. window leveling. Firstly, all cases were evaluated by an examiner with ten years of experience in musculoskeletal MRI diagnostics and age estimation (C.O.) and in order to determine the intraobserver-agreement a re-evaluation of all randomly rearranged cases was performed after a lapse of more than 6 months so as to prevent recall-bias. Secondly, an examiner with more than 20 years of experience in musculoskeletal MRI diagnostics and age estimation (V.V.) independently evaluated all cases for determining the interobserver-agreement. The evaluations were performed without knowledge of sex, age and earlier evaluations of the examined volunteers. Lastly, the assessed stages were then tested against the proven age.

### Low-field classification

The study group decided to use a PDw Dixon sequence. The rationale behind this decision was, that the Dixon technique could provide morphological information about osseous structures via the fat-only series and about watery structures via the water-only series, all in a single scan and with elevated sensitivity towards watery signals. After reviewing the acquired data, it became obvious that relevant landmarks of the distal radius’ physis were identifiable and therefrom a classification was construed that builds upon the synergistic use of the acquired images. The fat-only series is used to evaluate the osseous characteristics of the epiphysis and locate the physis or in later phases the physeal scar. Then, the water-only series is used for the purpose of detecting watery components within the examined osseous structures in the continuity of the physis. Thusly, it is possible to assess a stage by certain changes of the physeal ossification that are associated with the individual’s age. While stages 1–5 can already be differentiated solely in the fat-only sequence, the appearance of stages 5 and 6 are virtually identical in the fat-only sequence. In these stages, the water-only sequence is used to look for a hyperintense signal in the continuity of the epiphyseal scar. In stage 5, this hyperintense signal is present, while in stage 6 no such hyperintense signal can be found, marking the end of discernable morphological changes of the fusion as recognized by this classification. Furthermore, as the cohort’s lower bounds lay at 12 years of age, the acquired images can’t have captured the earliest developmental features of the physis. To signal awareness of this fact stage 1 was therefore left blank and the classification starts with stage 2.

**Fig. 1:**
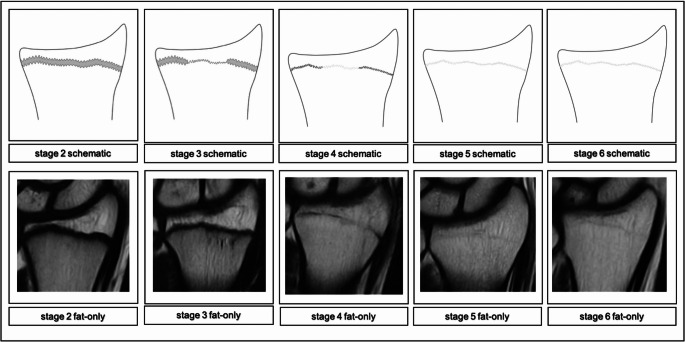
Schematic drawings for the stages in the PDw Dixon sequence fat-only series and examples (0.31T; non-contrast enhanced; coronal slice orientation); from left to right: male 14yrs, male 15yrs, male 17yrs, male 22 yrs, female 24yrs

**Fig. 2:**
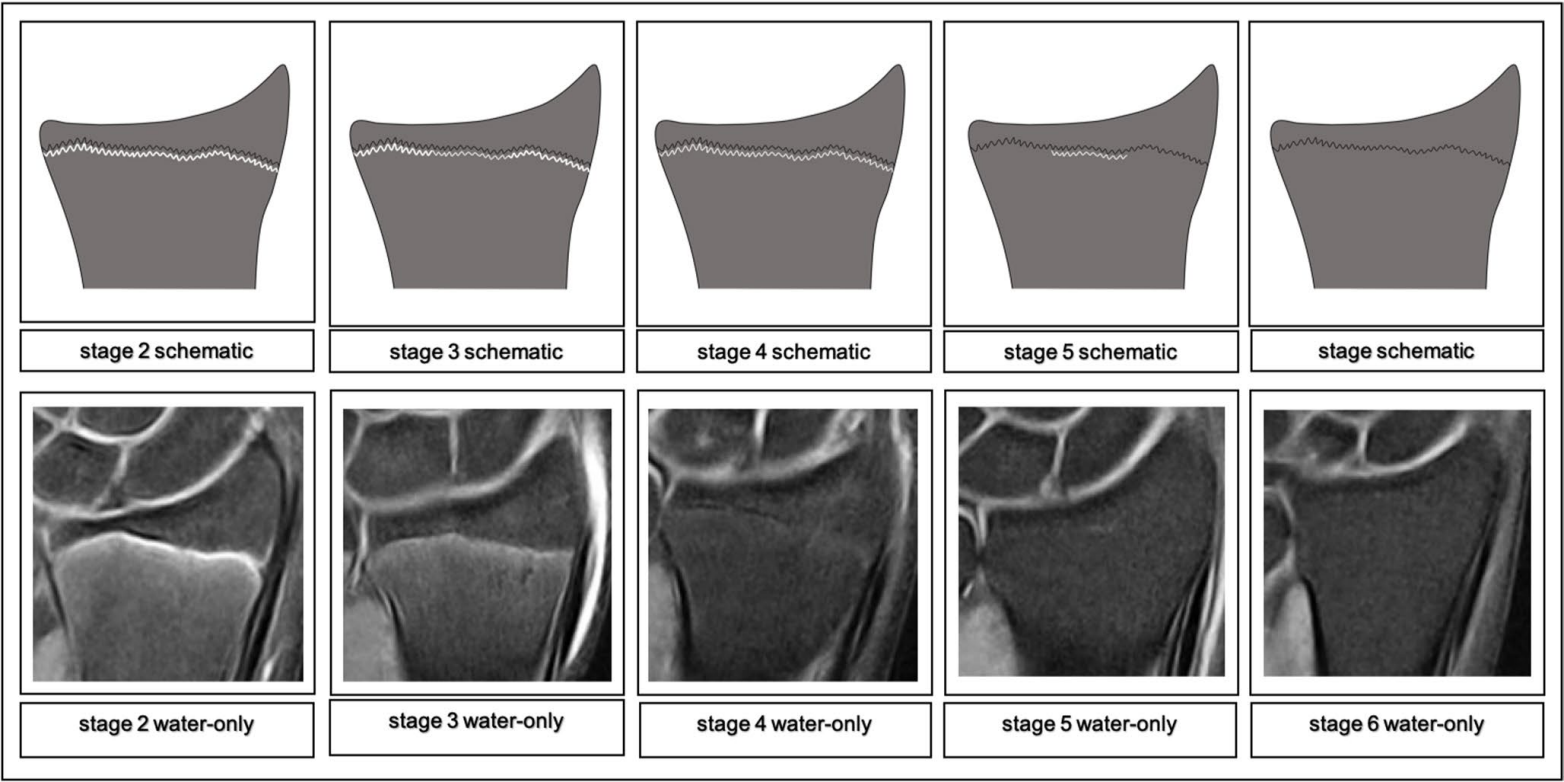
Schematic drawings for the stages in the PDw Dixon sequence water-only series and examples (0.31T; non-contrast enhanced; coronal slice orientation); from left to right: male 14yrs, male 15yrs, male 17yrs, male 22 yrs, female 24yrs

Corresponding schematics were produced for the distal radius’ physis accompanied by examples of the stages (see Figs. 1 and 2).

Following is a comprehensive description of the stages’ key characteristics in the fat-only series and the water-only series adapted to low-field imaging:

- Stage 2 In the fat-only series a continuous, broad band of low signal intensity is discernable. The band’s borders are smooth or serrated and towards the diaphysis the border may be blurry.
  The water-only series shows a thick, hyperintense line congruent to the band of the corresponding fat-only series with smooth or serrated borders. Towards the diaphysis the border may be blurry, forming a band. In the course of the band layers of high signal intensity may be discernable, that can be continuous or discontinuous and sporadically convening.
- Stage 3 In the fat-only series a discontinuous, broad band of low signal intensity is discernable. The band’s borders are serrated towards the epiphysis and the diaphysis. The band sporadically narrows, forming a single, thick serrated line of low signal intensity.
  The water-only series shows a thick line of high signal intensity in the course of the band of the corresponding fat-only series, that is segmentally tapering into a continuous, thin serrated line of high signal intensity.
- Stage 4 In the fat-only series a discontinuous, thick serrated line of low signal intensity between the epiphysis and the diaphysis is discernable with thinner segments of intermediate signal intensity.
  The water-only series shows a continuous, discontinuous or dotted, thin line of hyperintense signal in the continuity of the line of the corresponding fat-only series.
- Stage 5 In the fat-only series a continuous, thin serrated line of intermediate signal intensity between the epiphysis and the diaphysis is discernable.
  The water-only series shows a discontinuous or dotted, thin serrated line or spots of hyperintense signal in the course of the thin line of the corresponding fat-only series.
- Stage 6 In the fat-only series a continuous, thin serrated line of intermediate signal intensity between the epiphysis and the diaphysis is discernable. The water-only series shows no hyperintense signal in the course of the thin line of the corresponding fat-only series.

### Statistical analyses

Statistical analyses were conducted using IBM SPSS Statistics 28 for Mac OS X (release 25th May, 2021, Build 28.0.1.1, IBM Corporation). Minimum, maximum, mean ± standard deviation and median with lower and upper quartiles were defined for each stage of the classification to find the minimal ages. Intra- and interobserver-agreements were determined by calculating the kappa coefficients. Sex related differences in the stage-assessment across the ages were analyzed using the Mann-Whitney-U Test to determine their statistical relevance (*p* < 0.05, exact, two-tailed).

## Results

In a total of 647 cases it was possible to assess distinct stages. In three cases an assessment was not possible due to excessive movement artefacts.

### Observed minimum ages in the radius` distal epiphysis

In male individuals, the minimum ages for stages 2, 3, 4, 5, and 6 of the epiphyseal-diaphyseal fusion of the radius distal epiphysis were 12.00, 12.00, 14.50, 16.67 and 18.42 yrs. respectively (see Table 1).

**Table 1:** Metrics and derived values for male individuals

Stage	Number of cases	Minimum age	Maximum age	Mean age and standard deviation	Lower quartile; median; upper quartile
2	55	12.00	16.58	13.37 ± 1.00	12.63; 13.33; 14.03
3	57	12.00	19.50	15.49 ± 1.41	15.07; 15.25; 16.76
4	66	14.50	24.08	18.39 ± 2.34	17.11; 17.88; 18.51
5	112	16.67	24.92	21.15 ± 1.95	19.57; 21.08; 22.71
6	30	18.42	24.83	23.54 ± 1.31	21.32; 24.17; 24.19

In female individuals, the minimum ages for stages 2, 3, 4, 5, and 6 of the epiphyseal-diaphyseal fusion of the radius distal epiphysis were 12.08, 12.17, 12.75, 15.58 and 17.25 yrs. Respectively (see Table 2).

**Table 2:** Metrics and derived values for female individuals

Stage	Number of cases	Minimum age	Maximum age	Mean age and standard deviation	Lower quartile; median; upper quartile
2	35	12.08	14.67	13.07 ± 0.76	12.25; 12.75; 13.23
3	44	12.17	17.17	14.53 ± 1.19	13.88; 14.36; 15.40
4	85	12.75	24.17	17.52 ± 2.46	15.98; 17.17; 18.48
5	131	15.58	24.67	20.99 ± 2.29	18.89; 21.08; 22.46
6	32	17.25	24.92	22.53 ± 1.93	21.19; 22.71; 23.65

### Intra- and interobserver-agreement

After calculating Cohen’s kappa, we found a substantial intraobserver-agreement level (κ = 0.885) and a substantial interobserver-agreement level (κ = 0.848).

### Statistical differences of the sexes

The performed Mann-Whitney-U Test concerning the radius’ distal epiphysis implies significant sex-related differences for stage 3 (*p* < 0.01) and stage 4 (*p* < 0.01), but not for stage 2 (*p* < 0.162), stage 5 (*p* < 0.193) and stage 6 (*p* < 0.146).

## Discussion

The presented classification is to the best of our knowledge the first dedicated classification for age estimation in the living in a low-field MRI environment. It obviously draws from experiences with the Vieth-classification [19], which can be used in a similar fashion to evaluate images of long bones’ epiphyses acquired via high-field MRI. Due to similarities of the depiction of the long bones physis’ main features, these two classifications also share similarities, as they are based on morphological changes associated with the progression of the epiphyseal-diaphyseal fusion and are purely descriptive. However, when using low-field scanners, it is not possible to acquire the same sequences as Vieth et al., namely a T1w TSE (turbo spin echo) and a T2w TSE SPIR (signal presaturation by inversion recovery). Furthermore, the immediate low-field substitute sequences to those original sequences, a T1w SE (spin echo) and a T2w STIR (short tau inversion recovery), are not sufficiently sensitive towards watery components and would thereby likely have a detrimental influence on the stages’ age minima. Subsequently, the more proton sensitive PDw Dixon sequence was chosen, as it yields more signal intensity from watery structures (having a high density of hydrogen nuclei i.e. protons), while also minimizing scan time and movement artefacts, since the images are acquired and calculated from a single scan. Altogether, these changes appeared technically too distinct and would have required too many assumptions to apply the Vieth-classification, which ultimately led to the formulation of a dedicated low-field classification.

The results show a progression of stages when checked against the proven age. With decreasing numbers of cases of earlier stages (fading-out) at the lower bounds of the age bracket come increasing numbers of cases of late stages (fading-in) at the upper bounds of the age bracket which implies that we indeed documented age relevant characteristics.

When looking into the minimum ages of the results for each stage, we can see that a stage 5 in male volunteers constitutes a completed 16th year of life as the minimum age for that stage is 16.67 yrs. Likewise a stage 6 in male volunteers means achieved majority via a completed 18th year of life as the minimum age for that stage is 18.42 yrs. In female volunteers however, we may only constitute a completed 16th year of life as the minimum age for stage 6 is 17.25 yrs and thereby does not allow to determine achieved majority via the ultimate stage of the chosen classification.

Furthermore, it is important to note that the minimum ages at the lower bounds and the maximum ages at the higher bounds of the age bracket, i.e. 12 and 24 years, and all derived numbers are influenced by selection bias. The actual values for the respective minimum age should be assumed to be lower and for the maximum age to accordingly be higher, as it has already been pointed out by Vieth et al. [19].

### Limitations

Though relevant statistical measures concerning the chronological progression of the distal radius’ epiphysis’ ossification by means of low-field MRI could be presented, the number of examined individuals does not suffice to recommend the application of the presented method in practically applied forensic age estimation. To this end, a validation by further studies with higher case numbers is needed. A similar call for caution in a comparable study was priorly also given by Krämer et al. concerning a low margin for statistical errors in the application of a different classification [11].

Counter-intuitively, a limitation to volunteers of German nationality and thereby to mostly western Caucasian ethnicity is not hindering the applicability of its results to other nationalities or ethnicities. Earlier studies have shown that the major factor towards the progression of skeletal development is the socio-economic status [14–16]. As the socio-economic status of all our participants is comparatively high, we can conclude that their skeletal development would progress faster than the global average and thereby uniformly lead to lower minimum ages. This means that the application of the results would support the typical *in dubio pro reo* approach, as lower age minima will be in favor of examined persons in applied forensic age estimation in the living.
